# Early relief of osteoarthritis symptoms with a natural mineral supplement and a herbomineral combination: A randomized controlled trial [ISRCTN38432711]

**DOI:** 10.1186/1476-9255-2-11

**Published:** 2005-10-21

**Authors:** Mark JS Miller, Komal Mehta, Sameer Kunte, Vidyanand Raut, Jayesh Gala, Ramesh Dhumale, Anil Shukla, Hemant Tupalli, Himanshu Parikh, Paul Bobrowski, Jayesh Chaudhary

**Affiliations:** 1 Center for Cardiovascular Sciences, Albany Medical College, Albany, New York, USA; 2 Vedic Lifesciences, Mumbai, India; 3 Ashok Raut Orthopedic Hospital, Virarwest, India; 4 Bhagwati Hospital, Mumbai, India; 5 K.J. Somaiya Medical College & Hospital, Mumbai, India; 6 Santerra Pharmaceuticals, LLC, Raleigh, North Carolina, USA

## Abstract

**Background:**

This study was designed to determine if a natural mineral supplement, sierrasil, alone and in combination with a cat's claw extract (*Uncaria guianensis*), vincaria, has therapeutic potential in mild to moderate osteoarthritis of the knee.

**Methods:**

Patients (n = 107) with mild to moderate osteoarthritis of the knee were randomly assigned to one of 4 groups; high dose sierrasil (3 g/day), low dose sierrasil (2 g/day), low dose sierrasil (2 g/day) + cat's claw extract (100 mg/day) or placebo, administered for 8 weeks. Treatment was double blinded. Primary efficacy variables were WOMAC scores (A, B, C and total). Visual analog score (VAS) for pain, consumption of rescue medication (paracetamol), and tolerability were secondary variables. Safety measures included vital signs and laboratory-based assays.

**Results:**

Ninety-one of the 107 patients successfully completed the protocol. All four groups showed improvement in WOMAC and VAS scores after 8 weeks (p < 0.001), in all 3 groups receiving sierrasil the magnitude of benefits were greater vs. placebo (WOMAC Total 38–43% vs. 27%) but this was not statistically significant. In reference to baseline values sierrasil treated groups had a considerably faster onset of benefits. Placebo-treated individuals failed to show significant benefits at 4 weeks (11% reduction in total WOMAC). In contrast, after 1 or 2 weeks of therapy all the sierrasil groups displayed significant reductions in WOMAC scores (p < 0.05) and at week 4 displayed a 38–43% improvement. VAS was significantly improved at 4 weeks in all groups (p < 0.001) but was significantly greater in all sierrasil groups compared to placebo (p < 0.05). Rescue medication use was 28-23% lower in the herbomineral combination and high dose sierrasil groups although not statistically different from placebo (P = 0.101 and P = 0.193, respectively). Tolerability was good for all groups, no serious adverse events were noted and safety parameters remained unchanged.

**Conclusion:**

The natural mineral supplement, sierrasil alone and in combination with a cat's claw extract, improved joint health and function within 1–2 weeks of treatment but significant benefits over placebo were not sustained, possibly due to rescue medication masking. Sierrasil may offer an alternative therapy in subjects with joint pain and dysfunction.

## Background

Osteoarthritis is a painful and debilitating joint condition that affects hundreds of millions worldwide [1]. Despite the prevalence of the disease, current therapeutic options are not optimal. Pharmaceutical approaches to disease management include the popular non-steroidal anti-inflammatory class (NSAIDs) which block cyclo-oxygenase (COX). NSAIDS provide symptomatic relief [2] but do not abrogate the underlying disease process. Moreover, therapy has recently been dominated by the COX-2 specific class, which was designed to reduce unwarranted renal and gastrointestinal side-effects associated with non-specific COX inhibitors [3]. However, recent studies have revealed heightened cardiovascular risks [4,5], which may limit the application of the COX-2 specific class of NSAIDs.

Use of complimentary medicine as an alternative therapeutic approach is common in conditions associated with pain and discomfort and especially when local traditions support such approaches. Examples of complimentary medicines that have been proposed to offer benefits for osteoarthritis include acupuncture [6], nutraceuticals [7-9] and botanicals [10,11]. Additionally, various forms of physical therapy offer established non-pharmacologic benefits [12].

Of the nutraceutical approaches one of the best known and widely used is glucosamine (alone or in combination with chondroitin). Glucosamine and chondroitin are structural elements of cartilage and matrix. The therapeutic approach centers on the assumption that ingestion of large amounts of these matrix elements will assist in the replacement of the comparable material that is lost as a result of the catabolic process associated with inflammation. Recently, it has been suggested that the absorption of oral glucosamine is not sufficient for the chondroitin production and cartilage deposition [13]. There is limited evidence for a direct anti-inflammatory action of glucosamine [14]. Nevertheless by design it is not an approach that influences the disease process directly, but rather is thought to maintain cartilage architecture in the face of ongoing catabolic pathways. Hence, not surprisingly, the onset of action in those individuals for whom it does provide relief is commonly on the order of months after treatment initiation [15-17]. Studies as to the efficacy of glucosamine and chondroitin have produced variable results [18-21] suggesting that the benefits of this approach may have limitations.

Botanicals, especially those with redox-based actions are promising in the treatment of chronic inflammation because of their inherent disease modifying characteristics. Green tea catechins, especially epigallocatechin gallate (EGCG), have been shown to limit human cartilage degradation in vitro [22,23] and maintain joint architecture in an animal model [24]. This anti-inflammatory action is thought to be the result of inhibition of transcriptional events, particularly prevention of NF-*κ*B activation by cytokines and oxidants. NF-*κ*B is a critical transcription factor in chronic inflammation and is a desirable target for new therapeutics, including pharmaceutical development, as it regulates numerous genes that contribute to the inflammatory process [25-27]. Of particular note for joints NF-*κ*B regulates the production of matrix metalloproteases (MMPs) by chondrocytes [28]. During inflammation or injury chondrocytes release MMPs, which in turn degrade the cartilage matrix, releasing glycosaminoglycans and eventually, glucosamine. Haqqi and colleagues have elegantly demonstrated how EGCG can prevent MMP formation and cartilage degradation in explants of human cartilage stimulated by the pro-inflammatory cytokine, IL-1β [22] and reviewed the use of botanicals, in general, as arthritis therapeutics [11].

Sierrasil^® ^is a natural mineral product derived from the Sierra Mountains in the USA that has a cultural history of use to treat joint pain. We have recently demonstrated in the Haqqi model of human cartilage explants, that extracts of sierrasil reduced cartilage degradation in response to IL-1β, as well as nitric oxide production secondary to the induction of inducible nitric oxide synthase [29,30]. Increased chondrocyte production of nitric oxide is associated with catabolic activities and inhibitors of inducible nitric oxide synthase display anti-inflammatory properties [23,30-35]. While an action of sierrasil was not directly assessed on transcriptional events nevertheless, that is a likely target based on its ability to suppress IL-1β mediated events in human cartilage.

In the human cartilage explant study with sierrasil, co-administration of an extract of the botanical cat's claw (*Uncaria guianensis*, Vincaria^®^) complimented these actions. Indeed, this *Uncaria guianensis *extract is a remarkably potent inhibitor of NF-κB activity and tumor necrosis factor (TNFα) production [36-38], with clear cytoprotective actions [39] and has already successfully completed a placebo controlled trial for osteoarthritis of the knee [10]. Cat's claw is a vine indigenous to the Amazon Rainforest, that has a long history of use for joint pain and chronic inflammation [40].

Given that sierrasil was able to limit human cartilage degradation induced by IL-1β in vitro [29], it was determined that further study in human osteoarthritis was warranted. Further, it was postulated that based on the acute in vitro observations on cartilage protection and suppression of inflammatory events sierrasil may result in a clinical response that was expedient. Given the complimentary actions in vitro with cat's claw, a combination therapy was also evaluated. The goal was to determine if these approaches offered benefits in a safe manner, over the course of two months of treatment with a focus on rapid induction of benefits.

## Methods

### Research Design

This randomized, double-blind, placebo-controlled multi-center trial was approved by the Institutional Ethics Committee of K.J. Somaiya Medical College & Hospital, Mumbai, India and was in compliance with the Helsinki Declaration.

### Participants

Subjects were recruited from three centers – two private arthritis clinics and the K.J. Somaiya Medical College & Hospital, in Mumbai, India. Inclusion criteria were ambulatory, adult patients of either sex and greater than 20 years of age. Mild to moderate osteoarthritis was determined by radiological examination and ARA functional class II or III, and Kellgren Lawrence classification for knee osteoarthritis grade II or Grade III, and a baseline functional assessment of overall pain of at least 50 mm on a 100 mm Visual Analog Scale.

Exclusion criteria were osteoarthritis of grade I or IV (Kellgren Lawrence or ARA functional class), existence of other forms of arthritis, arthroscopy of either knee within the past year, administration of intra-articular steroids within the past 3 months or hyaluronic acid in the last 9 months, pregnancy or lactating women or women not taking adequate contraceptive measures, presence of any concomitant unstable disease or abnormality of any clinically relevant laboratory test, evidence of severe renal or hematologic disease or severe cardiac insufficiency, moderate to severe neuropathy, and unwillingness to come to regular follow-up visits for the length of the study.

A Fixed Allocation Randomization procedure was used to assign interventions to the participants with a pre-specified probability. Randomization was done in blocks of four, related to the number of treatment groups, and the total possibilities were 24. Treatment codes were held in a sealed manner by investigators in case of a serious adverse event. The allocation sequence was generated by the consulting statistician and subjects were assigned treatment according to this algorithm until subject 96 (total possibilities) and then the same cycle was repeated. Allocation of treatment according to this method was assigned by authors (SK, KM) along with monitoring of trial supplies, blindness, and adverse event monitoring. Research coordinators however, were not involved in any trial related activities to avoid bias.

For ease of presentation the 4 subject groups are given the following descriptors:

Group A – High dose sierrasil

Group B – Low dose sierrasil

Group C – Low dose sierrasil plus cat's claw extract

Group D – Placebo

A total of 107 subjects were recruited based on a planned recruitment of 25 subjects per group (total of 100). This recruiting estimate was based on results obtained in a trial which demonstrated efficacy with the cat's claw extract alone [10].

The sequence of the study involved an assessment of vital signs, radiological assessment of the affected knee and laboratory tests one week before baseline. Laboratory tests included: complete blood count, erythrocyte sedimentation rate, SGPT, serum creatinine, urine pregnancy test. At baseline the following were recorded – vital signs, WOMAC score (A, B, C and total) and VAS. This was repeated at weeks 1, 2, 4, 6, and 8 after treatment initiation along with monitoring of compliance and adverse event monitoring. At the conclusion of the study at week 8, laboratory tests were repeated.

Patients were considered drop-outs from the study if they went more than 4 days without medication or failed to report within the fifth day of a scheduled visit. Protocol deviation was characterized by not attending a scheduled visit by more than one day, skipping medication for an entire day, consuming other medications without consulting the investigator and not bringing the rescue medication or study medication bottles at the time of a scheduled visit.

Patients were not allowed to consume any NSAIDs, analgesics, cartilage supplements, calcium supplements, steroids, or other agents that may affect the outcomes of the study other than the rescue medication. Any medication taken by subjects for two months prior to the inclusion of the study, and whose intake was stabilized, was permitted and monitoring that dosing of these medications was not changed for the duration of the investigation.

### Treatments

The duration of treatment was 8 weeks, administered as 2 capsules twice a day taken orally with meals. The rescue medication was paracetamol (acetaminophen) as a single tablet of 500 mg, at a dosage that was not to exceed 4 tablets a day.

Sierrasil^® ^is a 100% natural composite, yet novel composite mineral (SM317) containing silicate minerals of calcium, magnesium, potassium, sodium and aluminum, among others. Sierrasil contains primarily 45.0% SiO2 (silica), 9.5% aluminum (as aluminum silicate), 5.9% iron (in several mineral forms), 1.3% calcium (in various forms), and other trace elements (Table 1). Sierrasil alone was administered in two treatment groups. The high dose group received a total of 3 g/day, and the low dose group 2 g/day. In addition, there was a separate treatment group where low dose sierrasil (2 g/day) was combined with a cat's claw extract, vincaria^® ^(*Uncaria guianensis*) at a dose of 100 mg/day. The relative components of major mineral sources in sierrasil are noted in Table 1, along with the expected daily intake at the doses of 2 and 3 grams.

**Table 1:** Mineral composition of Sierrasil and the expected intake at the low (2 g/day) and high (3 g/day) doses. Mineral bioavailability in the various forms has not been established but data on acid liberalization suggest that for some minerals the dose available for absorption may be as low as < 0.1%.

**Metal assays (ICP-MS)**	**Total Intake at 2 g/day dose**	**Total Intake at 3 g/day dose**
Aluminum	188 mg/day	282 mg/day
Antimony	< 0.006 mg/day	< 0.009 mg/day
Arsenic	0.026 mg/day	0.035 mg/day
Barium	2.0 mg/day	2.9 mg/day
Beryllium	< 0.002 mg/day	< 0.003 mg/day
Bismuth	< 0.06 mg/day	< 0.09 mg/day
Cadmium	0.0036 mg/day	0.0054 mg/day
Calcium	26 mg/day	40 mg/day
Carbon	0.60 mg/day	0.90 mg/day
Chromium	0.040 mg/day	0.060 mg/day
Chromium (VI)	< 0.00048 mg/day	< 0.00072 mg/day
Cobalt	0.017 mg/day	0.026 mg/day
Copper	0.074 mg/day	0.11 mg/day
Iron	119 mg/day	178 mg/day
Lead	0.013 mg/day	0.020 mg/day
Lithium	0.02 mg/day	0.03 mg/day
Magnesium	11 mg/day	17 mg/day
Manganese	0.29 mg/day	0.44 mg/day
Mercury	0.0012 mg/day	0.0017 mg/day
Molybdenum	0.002 mg/day	0.003 mg/day
Nickel	0.027 mg/day	0.040 mg/day
Phosphorous	3.8 mg/day	5.7 mg/day
Potassium	20 mg/day	30 mg/day
Selenium	0.0030 mg/day	0.0045 mg/day
Silica	899 mg/day	1349 mg/day
Silver	0.015 mg/day	0.022 mg/day
Sodium	< 12 mg/day	< 18 mg/day
Strontium	2.1 mg/day	3.2 mg/day
Sulphur	30 mg/day	45 mg/day
Thorium	< 0.010 mg/day	< 0.015 mg/day
Tin	< 0.02 mg/day	< 0.03 mg/day
Titanium	10 mg/day	15 mg/day
Uranium	< 0.12 mg/day	< 0.18 mg/day
Vanadium	0.34 mg/day	0.51 mg/day
Zinc	0.082 mg/day	0.12 mg/day
Zirconium	0.23 mg/day	0.35 mg/day

Cat's claw is an Amazonian vine whose bark has a long history of ethnomedical use for treating inflammation [38,40]. Commonly two species are used *Uncaria tomentosa *and *Uncaria guianensis*, and the Vincaria^® ^extract uses the *Uncaria guianensis *species, which was chosen as it is has been shown to be more potent as an anti-inflammatory agent [38], and is the only source of cat's claw with documented benefits in osteoarthritis [10]. The vincaria extract, which is a water based extraction, is devoid of oxindole alkaloids [38], present in *Uncaria tomentosa *that are thought to be immune enhancing [41,42], and therefore counter-productive for this application.

Sierrasil (SM317) was obtained from the manufacturer, Sierra Mountain Minerals, Inc. Bozeman, MT, USA  and Vincaria cat's claw (RN180) was obtained from Rainforest Nutritionals, Inc., Raleigh, NC, USA . Sierrasil and vincaria are produced under GMP conditions. Vincaria is standardized to a maximum level of oxindole alkaloids of 0.3 mg/g and an IC50 for TNF αinhibition of at least 1 ug/ml. Lactose was used as the placebo and as a filler for treatment group capsules to ensure that all treatments were uniform in size and color. Uniformity was maintained for all four treatment groups in terms of bottle filling, labeling, and packaging. Treatments were packaged in gelatin capsules and packed in wide mouthed white opaque plastic bottles with screw caps in a clean room. Investigators were provided with blinding chits having patient codes along with their treatment group (alphabetical code). In the case of a serious adverse event investigators were instructed to inform the monitors and then only unblind the treatment group of the subject in order to address needful treatment.

### Primary Efficacy Variable

The Western Ontario and McMaster Universities (WOMAC) Osteoarthritis Index is a disease-specific self-administered, health status measure that is widely accepted as reflective of osteoarthritis disease activity. The original index consists of 24 questions (5 pain, 2 stiffness and 17 physical functions). Individual question responses are assigned a score of between 0 (none) to 4 (extreme) and summed to form a score ranging from 0 (best) to 96 (worst). There are three sections to the WOMAC Score. Section A deals with the amount of *pain *(5 questions). Section B addresses the amount of *joint stiffness *(2 questions). Section C addresses aspects of *physical function *(17 questions).

In section C of the WOMAC Score 5 questions were addedfor a total of 22 questions. This score was then normalized to produce a total WOMAC score in a range of 0–100. Details of all questions included, per section, are found in Table 2. The addition of questions to the Section C component of WOMAC, which primarily addresses physical function, may be skewed the WOMAC total score away from pain and stiffness. Nevertheless, the pain and stiffness components were assessed individually, as well as collectively, to ensure that specific benefits could be ascertained as well as a modified global assessment.

**Table 2:** Modified WOMAC Questionnaire. Subjects responded with the following numerical assessment: 0 = none; 1 = slight; 2 = moderate; 3 = severe; 4 = extreme

**WOMAC A: Pain on**
- Walking
- Stair climbing
- Nocturnal
- Rest
- Weight bearing

**WOMAC B: Stiffness**
- In morning
- Stiffness occurring during the day

**WOMAC C: Level of difficulty performing the following functions:**
- Descending stairs
- Ascending stairs
- Rising from sitting
- Standing
- Bending to the floor
- Walking on flat
- Getting in/out of a car
- Going shopping
- Putting on socks-Rising from bed
- Taking off socks
- Lying in bed
- Getting in/out of bath
- Sitting
- Getting on/off toilet
- Heavy domestic duties
- Light domestic duties
- While working
- Sitting cross legged
- While cycling
- While driving vehicle
- While praying.

### Secondary Efficacy Variables

VAS – Visual Analog Score uses a 100 mm linear measure of pain status, with 0 representing no pain and 100 being unbearable pain. Patients marked on the linear scale the relevant amount of pain they were experiencing and the value was noted by the investigator.

Recovery, as assessed by investigator and physician, was characterized by 5 categories: Excellent – complete relief of symptoms; Good – partial relief of symptoms; Fair – minimal relief of symptoms; poor – no relief of symptoms; very poor – worsening of symptoms.

Tolerability was assessed by 3 categories. Good – no side effects; Fair – mild to moderate side effects; poor – severe side effects and withdrawal of therapy. Measurements of recovery and tolerability were performed at the end of the protocol (week 8).

Use of rescue medication, paracetamol (acetominophen) was addressed as a measure of both pain management and efficacy. The amount of rescue medication was only assessed in terms of total use at the conclusion of the study period. Rescue medication use was not assessed sequentially along with other variables.

### Data Quality Assurance

All investigators were informed of ICH-GCP guidelines, the quality of data and study execution was monitored by individuals independent of subject contact and treatment assessment.

### Statistical Analysis

Data was analyzed by an independent statistician using the following tests – ANOVA, paired and unpaired t tests, Bonferroni, Chi Square, Friedman and Wilcoxan tests as appropriate. The following software was used: SPSS 11.5, PEPI, EPI INFO 2000 and MS Excel. Statistical significance was taken at the 95% level (p < 0.05). Results are expressed as the mean ± SEM.

## Results

### Patient Randomization

Patient randomization was effective. Subject age was comparable in all groups (Table 3). Gender was predominantly female 73/107 (68.2%) vs. males 34/107 (31.8%). At entry 70% of subjects had ARAF-Class II and 30% ARAF-Class III. A similar distribution was observed for the Kellgren Lawrence Criteria for diagnosis, with 79% Grade 2 and 21% Grade 3 indicating that the majority of subjects had mild osteoarthritis of the knee. When the four treatment groups were compared there was no statistical difference in disease activity on entry – for ARAF-Class, Chi square = 0.586, p = 0.900; Kellgren Lawrence Criteria, Chi square = 0.690, p = 0.876.

**Table 3:** Baseline characteristics of treatment groups

	**Placebo**	**Group A**	**Group B**	**Group C**
**N completed (started)**	23 (29)	20 (25)	22 (24)	26 (29)
**Age (years)**	51.3 ± 1.0	50.5 ± 1.7	52.3 ± 1.8	52.1 ± 1.6
**Gender (F: M %)**	72:28	76:24	75:25	52:48
				
**ARA Functional Class II/III, %**	69 : 31	76 : 24	67 : 33	69 : 31
**Kellgren Lawrence Criteria Grade 2/3, %**	76 : 24	83 : 17	83 : 17	82 : 18
				
**WOMAC A**	8.7 ± 1.0	9.1 ± 1.2	8.0 ± 1.1	8.7 ± 1.0
**WOMAC B**	3.4 ± 0.5	3.5 ± 0.5	3.3 ± 0.4	3.3 ± 0.4
**WOMAC C**	36.4 ± 3.9	38.4 ± 4.0	34.7 ± 4.2	32.8 ± 4.3
**WOMAC Total**	48.6 ± 5.2	51.1 ± 6.4	46.4 ± 5.6	44.8 ± 5.5
				
**VAS**	72.9 ± 2.0	72.9 ± 3.0	70.1 ± 2.5	70.7 ± 2.4

Data expressed as a % of total or mean ± sem. Group A = High dose sierrasil, Group B = Low dose sierrasil, Group C = Low dose sierrasil + cat's claw extract. None of the entry assessments were significantly different amongst the four randomized groups.

### Primary Efficacy Variable – WOMAC

Baseline disease activity was comparable in all 4 treatment groups (Table 3). While all four groups displayed an improvement from baseline for WOMAC values (subsets and total) over the course of 8 weeks of treatment (p < 0.001), including placebo, the magnitude of these benefits were greater in all 3 sierrasil groups (A, B and C). It is of note that after 4 weeks of treatment the placebo treated group (D) was not significantly different from baseline for any of the WOMAC scores, yet for groups A, B and C there was a marked improvement over baseline (p < 0.001). Indeed, statistically significant benefits were evident with one week of treatment for many of the tests in sierrasil treated subjects (Tables 4, 5, 6, 7).

**Table 4:** Sequential WOMAC A Scores – Pain

**Group**	**Baseline**	**Week 1**	**Week 2**	**Week 4**	**Week 6**	**Week 8**
**Placebo**	8.7 ± 1.0	8.9 ± 1.1	8.5 ± 1.2	8.4 ± 1.2	6.8 ± 1.1 *	6.3 ± 1.2 *
**A**	9.1 ± 1.2	8.1 ± 1.2 *	7.5 ± 1.2 **	6.5 ± 1.2 **	6.4 ± 1.2 **	6.3 ± 1.1 **
**B**	8.0 ± 1.1	7.5 ± 1.1	6.9 ± 1.1 **	5.7 ± 1.0 **	5.6 ± 1.1 **	5.0 ± 0.9 **
**C**	8.7 ± 1.0	8.0 ± 1.0b	6.9 ± 0.9 ***	5.6 ± 0.8 ***	5.0 ± 0.8 ***	4.3 ± 0.7 ***

Data expressed as mean ± sem. Group A = High dose sierrasil (n = 20), Group B = Low dose sierrasil (n = 21), Group C = Low dose sierrasil + cat's claw extract (n = 25), placebo n = 22. All groups displayed a significant change over the 8 weeks of the study (p < 0.001, ANOVA). Using the Wilcoxan Matched-Pairs Signed-Rank test the following descriptors reflect statistical significance from baseline; * p < 0.05, ** p < 0.01, *** p < 0.001.

**Table 5:** Sequential WOMAC B Scores – Joint Stiffness

**Group**	**Baseline**	**Week 1**	**Week 2**	**Week 4**	**Week 6**	**Week 8**
**Placebo**	3.4 ± 0.5	3.6 ± 0.5	3.1 ± 0.5	3.0 ± 0.5	2.6 ± 0.5 *	2.4 ± 0.5 *
**A**	3.5 ± 0.5	3.0 ± 0.5 *	2.9 ± 0.6 *	2.4 ± 0.5 **	2.4 ± 0.5 **	2.3 ± 0.5 **
**B**	3.3 ± 0.4	2.9 ± 0.5 *	2.6 ± 0.5 **	2.1 ± 0.4 **	2.2 ± 0.5 **	1.9 ± 0.3 **
**C**	3.3 ± 0.4	3.2 ± 0.4	3.0 ± 0.4	2.1 ± 0.3 ***	2.0 ± 0.3 ***	1.6 ± 0.4 ***

Data expressed as mean ± sem. Group A = High dose sierrasil (n = 20), Group B = Low dose sierrasil (n = 21), Group C = Low dose sierrasil + cat's claw extract (n = 25), placebo n = 22. All groups displayed a significant change over the 8 weeks of the study (p < 0.001, ANOVA). Using the Wilcoxan Matched-Pairs Signed-Rank test the following descriptors reflect statistical significance from baseline; * p < 0.05, ** p < 0.01, *** p < 0.001.

**Table 6:** Sequential WOMAC C Scores – Physical Function

**Group**	**Baseline**	**Week 1**	**Week 2**	**Week 4**	**Week 6**	**Week 8**
**Placebo**	36.4 ± 3.9	37.0 ± 4.3	35.0 ± 4.4	32.0 ± 4.3	29.5 ± 4.5	26.6 ± 4.6 **
**A**	38.4 ± 4.9	32.4 ± 5.3	31.0 ± 5.1 **	26.1 ± 4.6 ***	26.3 ± 4.6 ***	23.1 ± 4.6 ***
**B**	34.7 ± 4.2	31.3 ± 4.4 *	28.7 ± 4.4 **	24.5 ± 3.8 ***	22.8 ± 3.9 ***	21.3 ± 2.9 ***
**C**	32.8 ± 4.3	31.1 ± 4.0	29.1 ± 4.0 *	24.3 ± 3.4 ***	21.7 ± 3.5 ***	19.7 ± 3.4 ***

Data expressed as mean ± sem. Group A = High dose sierrasil (n = 20), Group B = Low dose sierrasil (n = 21), Group C = Low dose sierrasil + cat's claw extract (n = 25), placebo n = 22. All groups displayed a significant change over the 8 weeks of the study (p < 0.001, ANOVA). Using the Wilcoxan Matched-Pairs Signed-Rank test the following descriptors reflect statistical significance from baseline; * p < 0.05, ** p < 0.01, *** p < 0.001.

**Table 7:** Sequential WOMAC Total – Summary

**Group**	**Baseline**	**Week 1**	**Week 2**	**Week 4**	**Week 6**	**Week 8**
**Placebo**	48.6 ± 5.2	49.0 ± 5.8	46.6 ± 5.9	43.5 ± 5.8	38.8 ± 5.9 *	35.3 ± 6.3 **
**A**	51.5 ± 6.4	43.5 ± 6.9	41.4 ± 6.7 **	35.0 ± 6.2 ***	36.2 ± 6.2 ***	31.8 ± 6.1 ***
**B**	46.4 ± 5.6	42.0 ± 5.9 *	38.6 ± 6.0 ***	32.6 ± 5.2 ***	31.5 ± 5.3 ***	28.5 ± 3.9 ***
**C**	44.8 ± 5.5	42.3 ± 5.2	39.0 ± 5.1 **	32.0 ± 4.4 ***	28.7 ± 4.5 ***	25.6 ± 4.4 ***

Data expressed as mean ± sem. Group A = High dose sierrasil (n = 20), Group B = Low dose sierrasil (n = 21), Group C = Low dose sierrasil + cat's claw extract (n = 25), placebo n = 22. All groups displayed a significant change over the 8 weeks of the study (p < 0.001, ANOVA). Using the Wilcoxan Matched-Pairs Signed-Rank test the following descriptors reflect statistical significance from baseline; * p < 0.05, ** p < 0.01, ** p < 0.001. Note the p values for groups A and C approached significance at week one (p = 0.051 and 0.053).

Specifically, for group A (high dose) WSA, WSB and WST were significantly improved at week 1 (p < 0.05). At week 2 for group A, all WOMAC scores were significantly improved (p < 0.05). For group B (low dose), WSB, WSC, and WST were significantly improved at week 1 (p < 0.05). After 2 weeks of therapy in group B, all WOMAC scores were significantly improved (p < 0.01). For group C (low dose sierrasil + cat's claw extract) WSA was significantly improved from baseline (p < 0.01) and by week 2 all WOMAC scores except WSB were improved (p < 0.05).

**Figure 1:**
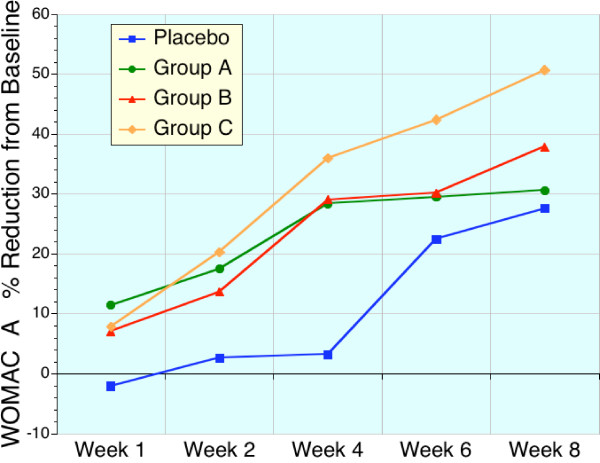
Sequential changes in WOMAC A (pain) scores expressed as a percentage of baseline values. Placebo (blue, n = 22), high dose sierrasil (green, n = 20), low dose sierrasil (red, n = 21) and low dose sierrasil + cat's claw extract (orange, n = 25) all demonstrated a time dependent improvement in the pain indices of WOMAC A. However, in the placebo group this was delayed until the last month of the study. All sierrasil treated groups displayed a faster onset of action.

**Figure 2:**
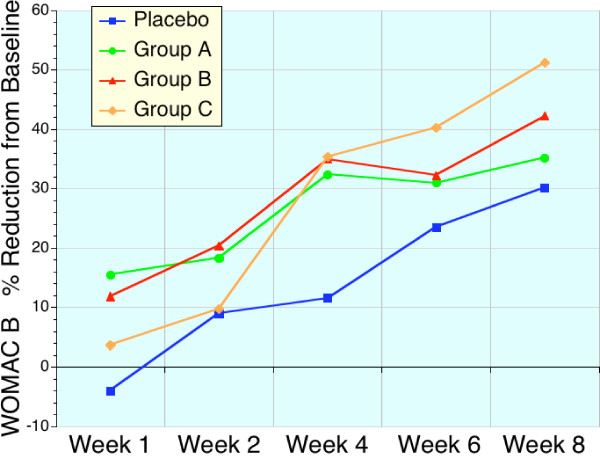
Sequential changes in WOMAC B (stiffness) scores expressed as a percentage of baseline values. Placebo (blue, n = 22), high dose sierrasil (green, n = 20), low dose sierrasil (red, n = 21) and low dose sierrasil + cat's claw extract (orange, n = 25) displayed a time dependent improvement in WOMAC B scores, measuring stiffness, over the course of the study. A trend for a faster onset of improvement was evident in all sierrasil treated groups when compared to placebo controls.

**Figure 3:**
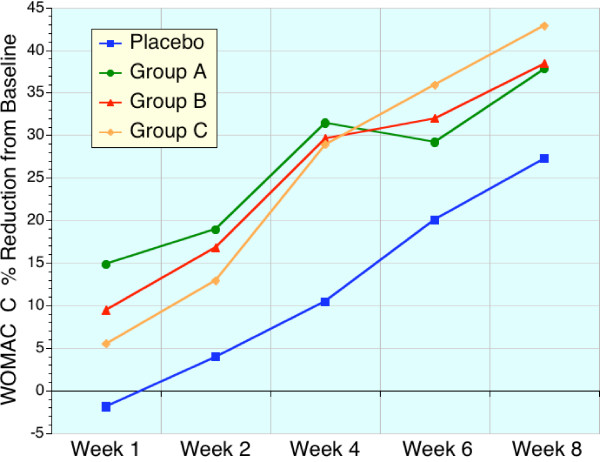
Sequential changes in WOMAC C (physical activity) scores expressed as a percentage of baseline values. Placebo (blue, n = 22), high dose sierrasil (green, n = 20), low dose sierrasil (red, n = 21) and low dose sierrasil + cat's claw extract (orange, n = 25) displayed a time dependent improvement in physical activity and function scores (WOMAC C). A trend for a faster onset of benefits was evident in all sierrasil treated groups versus placebo.

**Figure 4:**
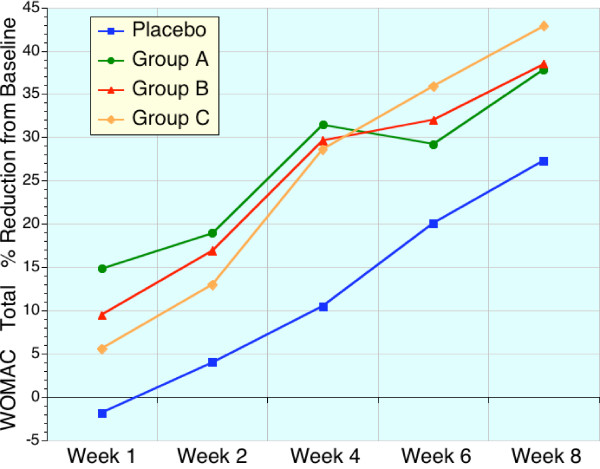
Sequential changes in WOMAC total scores expressed as a percentage of baseline values. Placebo (blue, n = 22), high dose sierrasil (green, n = 20), low dose sierrasil (red, n = 21) and low dose sierrasil + cat's claw extract (orange, n = 25) displayed a time dependent improvement in total WOMAC Scores. There was a trend for a faster onset of action in all sierrasil treated groups when compared to placebo responses.

These early benefits are readily apparent in Table 4, 5, 6, and Figures 1, 2, 3, 4 which displays the percentage changes from baseline for WOMAC A, WOMAC B, WOMAC C, and WOMAC total respectively. However, when the test groups were compared to placebo, the magnitude of these differences were not significant for the majority of time points (Friedman test).

### Secondary Efficacy Variables – VAS

**Figure 5:**
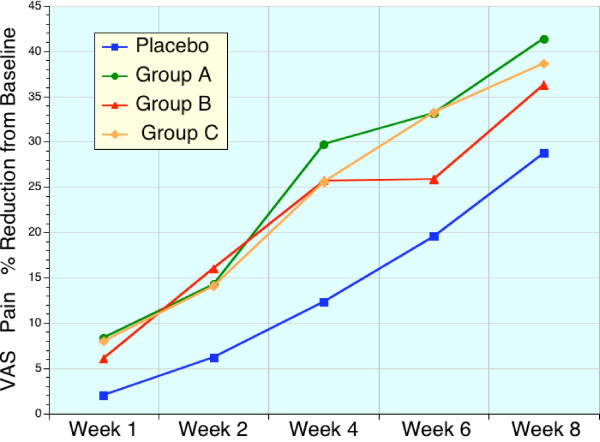
Sequential changes in VAS (pain) expressed as a percentage of baseline values. Placebo (blue, n = 22), high dose sierrasil (green, n = 20), low dose sierrasil (red, n = 21) and low dose sierrasil + cat's claw extract (orange, n = 25) displayed a time dependent improvement in VAS scores for pain. However, there was a trend for a faster onset of benefits in the sierrasil treated groups compared to placebo.

The VAS, a pain assessment, was significantly improved in all 4 treatment groups at the conclusion of the study (week 8, p < 0.001), Table 8. The placebo group (D) also displayed a significant improvement in VAS at 4 weeks as did groups A-C (P < 0.01) although, the magnitude of VAS reductions in these sierrasil groups (A-C) at week 4 were significantly greater than placebo (p < 0.05). However, the greater response in groups A-C vs. placebo, was not statistically significant at week 8 or other time points. Changes in VAS expressed as a percentage of change from baseline are shown in Figure 5.

**Table 8:** Sequential VAS – Pain Index

**Group**	**Baseline**	**Week 1**	**Week 2**	**Week 4**	**Week 6**	**Week 8**
**Placebo**	72.9 ± 2.1	71.4 ± 2.7	68.4 ± 3.2	63.9 ± 3.3 **	58.6 ± 3.9 **	52.0 ± 4.7 ***
**A**	72.9 ± 3.0	66.8 ± 3.3	62.5 ± 4.7 **	51.2 ± 5.0 ***	49.7 ± 5.1 ***	42.7 ± 5.2 ***
**B**	70.1 ± 2.5	65.8 ± 3.2	58.9 ± 3.8 **	52.0 ± 4.7***	51.9 ± 5.0 ***	44.6 ± 4.9 ***
**C**	70.7 ± 2.4	65.0 ± 2.6	60.7 ± 2.9 **	52.7 ± 2.5 ***	47.2 ± 3.4 ***	43.4 ± 3.8 ***

Data expressed as mean ± sem. Group A = High dose sierrasil (n = 20), Group B = Low dose sierrasil (n = 21), Group C = Low dose sierrasil + cat's claw extract (n = 25), placebo n = 22. All groups displayed a significant change over the 8 weeks of the study (p < 0.001, ANOVA). Using a Wilcoxan matched pairs Signed-Rank test from baseline; * p < 0.05, ** p < 0.01, *** p < 0.001.

### Secondary Efficacy Variable – Rescue Medication

**Figure 6:**
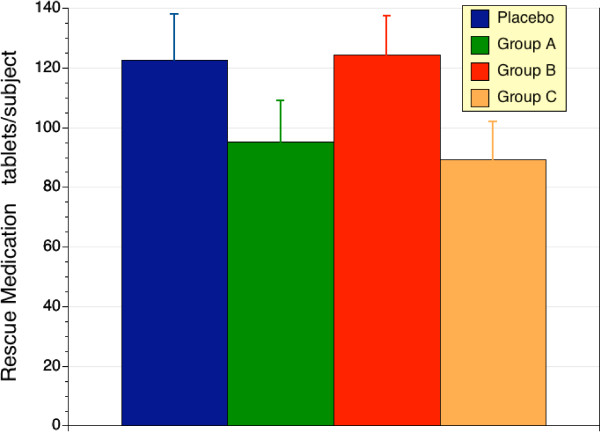
Consumption of rescue medication (paracetamol) for the study duration. Results for placebo (blue column, n = 19), high dose sierrasil (green column, n = 19), low dose sierrasil (red column, n = 20) and low dose sierrasil + cat's claw extract (orange column, n = 23) groups are expressed as mean ± sem. There was no significant difference in rescue medication use between the various groups, although a trend for less paracetamol consumption was evident in both the high dose sierrasil and low dose + cat's claw groups which approached significance (p = 0.119 and p = 0.101 vs placebo respectively).

Paracetamol was used as a rescue medication with dosing limited to 4 × 500 mg per day. There were no significant differences in the use of paracetamol in the 4 treatment groups (Figure 6), although some trends are clear. In group A, there was a 23% lower use of paracetamol (p = 0.193 v placebo). For group C there was a 28% reduction in paracetamol use, which approached significance when compared to placebo (p = 0.101) or low dose sierrasil alone (p < 0.055). The consumption of rescue medication was not assessed at sequential timepoints. Furthermore, the trend for greater consumption of rescue medication in the placebo and low dose sierrasil groups may have masked the ability to determine significant differences between groups in terms of the magnitude of pain and discomfort relief.

### Secondary Efficacy Variables – Global Assessments and Tolerance

Two global assessments to treatment were determined – patient self assessment and the investigator's assessment. For both perspectives 49% responded that the response was good to excellent, however there was no statistical difference between treatment groups Patient's response: Chi square = 8.118, p = 0.776.

Investigator's response: Chi square = 4.336, p = 0.888

Tolerance was reported as "good" by 94/95 subjects. The one subject that reported a poor tolerance received high dose sierrasil (group A).

### Safety Variables – Laboratory

All laboratory tests were unchanged from baseline to week 8 for all groups with the following exceptions.

(1) Group A hemoglobin was increased from 12.0 ± 0.2 to 12.5 ± 0.3, p < 0.05

(2) Group B lymphocyte levels increased from 30.3 ± 1.8 to 34.3 ± 1.8, p < 0.05

These changes, while statistically significant, were not considered as indicative of an adverse response based on values observed in the other groups. A summary of these safety variables is depicted in Table 9.

**Table 9:** Laboratory-based evaluations of safety

**Test**	**Placebo **Wk. 0	**Placebo **Wk. 8	**A **Wk. 0	**A **Wk. 8	**B **Wk. 0	**B **Wk. 8	**C **Wk. 0	**C **Wk. 8
**Neu**	62.0 ± 1.7	64.2 ± 2.1	59.3 ± 2.4	58.6 ± 2.3	64.7 ± 1.6	61.5 ± 1.6	61.6 ± 1.5	63.0 ± 1.7
**Bas**	0	0	0	0	0	0	0	0.3 ± 0.3
**Eos**	3.6 ± 0.8	3.6 ± 0.6	4.2 ± 0.5	3.7 ± 0.9	4.0 ± 0.6	4.8 ± 1.5	3.9 ± 0.5	4.4 ± 0.5
**Lym**	34.0 ± 2.0	31.7 ± 2.5	35.7 ± 2.5	35.2 ± 1.8	30.3 ± 1.8	34.3 ± 1.8*	33.4 ± 1.7	31.8 ± 1.8
**Mon**	0.8 ± 0.3	0.6 ± 0.2	1.0 ± 0.2	0.8 ± 0.3	0.9 ± 0.2	0.7 ± 0.2	1.1 ± 0.2	0.7 ± 0.2
**WBC**	8052 ± 417	8260 ± 499	7994 ± 514	8037 ± 655	8750 ± 476	7857 ± 478	8160 ± 308	7815 ± 459
**RBC**	3.98 ± 0.09	4.11 ± 0.11	4.26 ± 0.09	4.41 ± 0.12	4.33 ± 0.11	4.31 ± 0.10	4.39 ± 0.12	4.30 ± 0.10
**HB**	12.0 ± 0.2	12.4 ± 0.4	12.0 ± 0.2	12.5 ± 0.3*	12.2 ± 0.3	12.1 ± 0.3	12.3 ± .2	12.2 ± 0.3
**ESR**	29.0 ± 3.6	32.4 ± 6.0	35.0 ± 3.5	30.5 ± 4.8	31.7 ± 4.2	40.7 ± 6.2	26.5 ± 3.6	22.2 ± 3.2
**SGPT**	28.8 ± 1.9	27.7 ± 2.4	23.6 ± 1.9	29.3 ± 4.5	24.5 ± 1.6	25.6 ± 1.7	24.9 ± 1.1	26.4 ± 3.3
**CRE**	0.98 ± 0.04	0.97 ± 0.05	1.4 ± 0.4	0.9 ± 0.04	1.7 ± 0.7	1.0 ± 0.03	1.67 ± 0.69	1.01 ± 0.05

Data expressed as a mean ± sem. Group A = High dose sierrasil (n = 20), Group B = Low dose sierrasil (n = 21), Group C = Low dose sierrasil + cat's claw extract (n = 25), placebo n = 22.

Neu = neutrophils %, Bas = basophils %, Eos = eosinophils %, Lym = lymphocytes %, Mon = monocytes %, WBC = white blood cells per mm^3^, RBC = red blood cells 10^6 ^× mm^3^, HB = hemoglobin gm/dl, ESR = erythrocyte sedimentation rate mm, SGPT = IU/L, CRE = creatinine mg/dl. Using unpaired t test, the following descriptors describe a significant difference from baseline, * p < 0.05.

### Safety Variables – Vital Signs

Blood pressure (systolic and diastolic), respiration rate and pulse rate were vital signs that were monitored. Using ANOVA there was no change in these variables from screening for the study, at the 5 intermediate evaluation points and the study's conclusion at 8 weeks, with the following exceptions.

(1) Pulse rate declined in Group B (p < 0.05) from a screening value of 81.4 ± 1.5 to 76.2 ± 1.2 beats per minute at week 8.

(2) Respiration rate declined in Group C (p < 0.05) from a screening value of 19.3 ± 0.6 to 18.9 ± 0.4 breaths per minute

Data for baseline vital sign values and again at week 8 are depicted in Table 10. Data for screening and the intervening assessments at weeks 1, 2, 4 and 6 are not included for simplicity.

**Table 10:** Vital Signs

**Vital Sign**	**Placebo Wk 0**	**Placebo Wk 8**	**A Wk. 0**	**A Wk. 8**	**B Wk. 0**	**B Wk. 8**	**C Wk. 0**	**C Wk. 8**
**Pulse Rate**	80.3 ± 1.3	76.9 ± 1.1	79.1 ± 1.3	74.7 ± 0.9	80.7 ± 1.3	76.1 ± 1.2	78.6 ± 0.9	76.6 ± 0.9
**Systolic BP**	128 ± 1	130 ± 2	129 ± 2	130 ± 3	132 ± 2	129 ± 3 *	129 ± 1	128 ± 2
**Diastolic BP**	83 ± 1	86 ± 1	82 ± 1	82 ± 1	83 ± 1	82 ± 1	80 ± 1	80 ± 2
**Respiration Rate**	19.5 ± 0.7	19.9 ± 0.7	19.2 ± 0.6	18.9 ± 0.4	19.7 ± 0.7	19.4 ± 0.8	20.2 ± 0.5	18.5 ± 0.5 *

Data expressed as a mean ± sem. Units of measure were: Pulse rate (beats per min), systolic and diastolic blood pressure (mm Hg), respiration rate (breaths per minute). Group A = High dose sierrasil (n = 20), Group B = Low dose sierrasil (n = 21), Group C = Low dose sierrasil + cat's claw extract (n = 25), placebo n = 22. Pulse rate and respiration rate are given as the rate per minute and blood pressure is expressed in mm Hg. Using unpaired t test, the following descriptors describe a significant difference from baseline, *p < 0.05.

## Discussion

The purpose of this study was to determine if the natural mineral supplement, sierrasil, would relieve the symptoms of mild to moderate osteoarthritis of the knee in a safe manner. The approach included two doses of the mineral supplement to encompass the anecdotal clinical experience, and to evaluate the inclusion of a botanical extract, cat's claw (vincaria), which had previously been shown to be effective in treating osteoarthritis [10]. Using a randomized, double-blind placebo-controlled multi-center design, it is clear that this mineral supplement is indeed safe. Treatments were efficacious, particularly compared to baseline conditions, but there were clear difficulties in determining a sustained disassociation from placebo. In all sierrasil treated groups there was a significantly faster onset of benefits from initial values (evident from week 1 to 2) compared to placebo (first evident at week 6) but at the conclusion of the study differences between groups was not significant.

While it is of interest that the sierrasil that provided early relief of symptoms the inability to establish sustained significant differences from placebo poses limitations on interpretation. In part this dilemma is the result of the small study group size in this preliminary clinical evaluation. Additionally, an unexpected sharp improvement in primary and secondary assessments in the placebo group at weeks 6 & 8 contributed to the study's limitations. While clearly not in the instructions, subjects may have had expectations that all potential treatments in the randomized protocol would provide benefits and this may account for the placebo response. However, if this were the case one may expect that this placebo effect would be continuous as opposed to an exaggerated response that was observed in the last month of a two month study.

Another important consideration is a potential masking effect of rescue medication use. Rescue medication use was greater in placebo and low dose sierrasil groups, and this may have masked differences between the positive benefits related to treatment and placebo. Total consumption of rescue medication was determined and so it is not possible to link changes in rescue medication to perceived changes in disease activity on a weekly or monthly basis.

With the sierrasil test groups significant reductions in the baseline values of the primary efficacy variable, WOMAC, were evident as early as week 1 with steady improvements with continued administration (Figs. 1, 2, 3, 4). In contrast, placebo treated subjects did not report significant benefits from baseline until week 6. This early onset of benefits is not inconsistent with the in vitro studies demonstrating the protection of human cartilage degradation induced by IL-1β, which was prevented by acute exposure to sierrasil [25]. These human cartilage explant studies also demonstrated that the activation of nitric oxide production, a catabolic pathway [30-32], was attenuated by sierrasil. However, the present study does not directly assess whether protection of against cartilage degradation was associated with the therapies, nor is it likely that a substantial change in joint architecture would occur in this timeframe.

Of note, co-administration of the *Uncaria guianensis *extract, vincaria, was also chondroprotective in vitro [29] and associated with a rapid onset of benefits in osteoarthritis as noted in a separate double blind placebo controlled study [10]. Cat's claw has considerable data demonstrating that it is an effective inhibitor of transcription via NF-κB [36] and this formulation is a quite potent inhibitor of tumor necrosis factor [37,38]. Thus, both sierrasil and vincaria have the potential to act as disease modifying agents in osteoarthritis although only safety and symptomatic relief were the focal issues of this preliminary clinical study.

As this mineral supplement is relatively unknown it was important to evaluate safety as well as efficacy. In this 2 month study there were no changes in various clinical and laboratory measures of safety. The study design included an evaluation of mineral supplement dose (2 vs. 3 g/day) and the herbomineral combination. The reason for evaluating these somewhat similar doses reflects the anecdotal clinical experience with these doses, which brought suggestions that the higher dose necessitated a more rigorous assessment. There was little difference between these groups although a better defined week 1 and 2 responses with the high dose were evident. The herbomineral combination produced a greater percentage reduction in WOMAC scores and both groups were associated with reduced consumption of rescue medications. This suggests that these approaches provided additional value but a definitive statistical difference to advocate a higher dose or the herbomineral combination was not achieved. However, with a greater subject enrolment these trends would likely have reached significance.

VAS, as an index of pain, was responsive to both treatment as well as placebo. While there were significant differences at 1 month between mineral supplement treatments and placebo this was not statistically evident at 2 months. Indeed there was a trend for an exaggerated placebo effect for many efficacy variables from week 4 to week 8.

VAS is regarded as being a less sensitive index of disease activity than the WOMAC scores, which assess pain as well as stiffness and physical activity/function. The similar trends for earlier symptomatic relief in sierrasil treated subjects in the all three WOMAC subsets, as well as VAS pain, within the first month was noticeable and readily distinguishable from placebo treated individuals (Figs 1, 2, 3, 4, 5). Indeed, the similar trends in these three treatment groups when taken together suggest that this therapeutic approach has a clear early onset of benefits.

The mechanisms by which this natural mineral supplement achieves these actions and benefits is unclear. The study in human explants indicates that it may affect transcriptional events, as indicated by the reduced production of nitric oxide in response to IL-1β. Increased production of nitric oxide under these circumstances is attributed to the expression of inducible nitric oxide synthase [31,35]. A decade ago we defined that this nitric oxide isoform promoted chronic inflammation [34,35] and inhibitors alleviate numerous inflammatory conditions including arthritis [30].

The protection of matrix degradation in response to IL-1 by sierrasil may reflect a reduction in matrix metalloprotease (MMP) production (also transcriptionally regulated in response to IL-1β) or perhaps a direct interference in the activity of MMPs. There is no evidence for the later but is speculated based on the mineral/metal content of sierrasil and the requirement of metals as catalysts in MMPs. Studies by the manufacturer on the acid liberalization of minerals from sierrasil suggest that the majority of the minerals within sierrasil are tightly bound and are not freely bioavailable (data not shown). Additionally, while the ingestion of supplemental minerals may alter the basal nutritional status of the subjects the literature does not provide a clear link between a nutrition-based action of minerals and an effective anti-arthritic therapy [43]. It is speculated that a micromineral action on gene expression or direct interaction with MMPs may be an alternative action to consider.

A critical issue is how to place these findings in a therapeutic perspective. Osteoarthritic patients, and their healthcare providers, are deeply concerned with the recent documentation of an increased risk for cardiovascular disease and stroke with COX-2 inhibitors [4,5], as well as the significant gastro-intestinal, renal complications and premature deaths associated with non-selective COX inhibitors [3]. With the appreciation that the NSAID class provides symptomatic relief rather that abrogating the disease process [2], there is a great need for alternatives.

Despite the potential of redox-based botanicals as anti-inflammatory agents [11], the most commonly used natural product approach to the management of osteoarthritis is glucosamine and chondroitin [43]. While early studies suggested excellent albeit slow-onset responsiveness [15-17], more recent studies have suggested that the benefits offered by glucosamine and chondroitin may have limitations or be variable in nature [13,18-21]. One recent study suggested that continued use of glucosamine may be unwarranted, even if there were initial benefits [44]. Clarification as to therapeutic potential of this approach is likely to occur when the results of the Glucosamine Arthritis Intervention Trial, with 1588 subjects from 13 centers randomized to 5 treatment groups.

While sierrasil was noted to produce early benefits but it was not clearly established that sustained actions were indistinguishable from placebo. Similar issues have been raised with glucosamine and chondroitin, as well as cetylated fatty acids, where there is some evidence that after 2 months of therapy there are reductions in pain and flexibility but no benefits related to physical function/activity [9].

## Conclusion

In summary, the natural mineral supplement sierrasil, alone and in combination with an extract of the Amazonian medicinal plant, cat's claw (*Uncaria guianensis*), provide a rapid relief of osteoarthritis symptoms. The benefits were evident within a week, and associated with an excellent safety profile. However, a lack of discrimination between treatment and placebo, especially with sustained administration, limits the assessment sierrasil's role in the treatment of osteoarthritis. Nevertheless, as alternative approaches to the management of osteoarthritis are desirable, sierrasil may offer a valuable option for some subjects.

## Competing interests

MJSM has an equity interest in Santerra Pharmaceuticals, LLC & Rainforest Nutritionals, Inc.

PB has an equity interest in Santerra Pharmaceuticals, LLC & Rainforest Nutritionals, Inc.

## Authors' contributions

MJSM assisted in study design, data analysis and manuscript preparation.

VR contributed to patient recruitment, evaluation and study execution

JG contributed to patient recruitment, evaluation and study execution

RD contributed to patient recruitment, evaluation and study execution

KM assisted in data management, study design monitoring and site communication, laboratory monitoring and manuscript drafting.

SK contributed to study design, GCP monitoring and document planning

AS contributed to site selection and monitoring, protocol compliance and laboratory monitoring and control

HT contributed to protocol and case report form writing.

HP contributed to study design, project planning, quality assurance and manuscript preparation

PB contributed to study design and manuscript preparation

JC contributed to study design, project planning, quality assurance and manuscript preparation.
